# Peculiar optical properties of bilayer silicene under the influence of external electric and magnetic fields

**DOI:** 10.1038/s41598-018-36547-1

**Published:** 2019-01-24

**Authors:** Thi-Nga Do, Godfrey Gumbs, Po-Hsin Shih, Danhong Huang, Chih-Wei Chiu, Chia-Yun Chen, Ming-Fa Lin

**Affiliations:** 1 Institute of Physics, Academia Sinica, Taipei, 11529 Taiwan; 2 Department of Physics and Astronomy, Hunter College of the City University of New York, 695 Park Avenue, New York, 10065 USA; 3 Donostia International Physics Center (DIPC), P de Manuel Lardizabal, 4, 20018 San Sebastian, Basque Country Spain; 4 Department of Physics, National Cheng Kung University, Tainan, Taiwan; 5 US Air Force Research Laboratory, Space Vehicles Directorate, Kirtland Air Force Base, New Mexico, 87117 USA; 6 Department of Physics, National Kaohsiung Normal University, Kaohsiung, Taiwan; 7 Department of Materials Science and Engineering, International Curriculum for Advanced Materials Program (iCAMP), National Cheng Kung University, Tainan, Taiwan; 8 Quantum Topology Center, National Cheng Kung University, Tainan, Taiwan; 9 Hierarchical Green-Energy Materials Research Center, National Cheng Kung University, Tainan, Taiwan

## Abstract

We conduct a comprehensive investigation of the effect of an applied electric field on the optical and magneto-optical absorption spectra for AB-bt (bottom-top) bilayer silicene. The generalized tight-binding model in conjunction with the Kubo formula is efficiently employed in the numerical calculations. The electronic and optical properties are greatly diversified by the buckled lattice structure, stacking configuration, intralayer and interlayer hopping interactions, spin-orbital couplings, as well as the electric and magnetic fields (${E}_{z}\hat{z}$ $\&$ ${B}_{z}\hat{z}$). An electric field induces spin-split electronic states, a semiconductor-metal phase transitions and the Dirac cone formations in different valleys, leading to the special absorption features. The *E*_*z*_-dependent low-lying Landau levels possess lower degeneracy, valley-created localization centers, peculiar distributions of quantum numbers, well-behaved and abnormal energy spectra in *B*_*z*_-dependencies, and the absence of anti-crossing behavior. Consequently, the specific magneto-optical selection rules exist for diverse excitation categories under certain critical electric fields. The optical gaps are reduced as *E*_*z*_ is increased, but enhanced by *B*_*z*_, in which the threshold channel might dramatically change in the former case. These characteristics are in sharp contrast with those for layered graphene.

## Introduction

Layered condensed-matter systems, with varied physical properties and many potential device applications, have so far attracted a great deal of experimental and theoretical attention^1–23^. Recently, theoreticians have developed reliable models in order to explore basic physical properties of newly discovered two-dimensional (2D) materials, especially for their electronic and optical properties when external electric and magnetic fields are applied. Few-layer 2D materials are the main focus, mainly because of their eclectic lattice symmetries, their nano-scaled thickness, and their inherently unique interactions. Group-IV and -V 2D systems, which have been successfully fabricated in laboratory conditions, include graphene^1–3^, silicene^4–13^, germanene^14,15^, tinene^16,17^, phosphorene^18–20^, antimonene^21^, and bismuthene^22,23^. These systems are expected to play crucial roles in basic and applied sciences, in which the rich and unique properties are worthy of a systematic investigation. In the present work, we concentrate our efforts to achieve a full understanding of the optical absorption spectra of bilayer silicene, being closely related to the electronic properties in the presence/absence of electric and magnetic fields. The generalized tight-binding model, combined with the dynamic Kubo formula, are fostered for exploring the diversified essential properties thoroughly. All the intrinsic interactions and the external fields are taken into consideration simultaneously. The distinct behaviors of the energy bands, density of states (DOS), quantized Landau levels (LLs), spatial magneto-wave functions, and absorption spectra are discussed adequately.

Previous investigations have demonstrated that 2D group-IV structures exhibit particularly exceptional properties. In this regard, graphene has an sp^2^-bonding planar structure, whereas silicene and germanene have buckled structures with a slightly mixed sp^2^-sp^3^ chemical bonding^2,13,15^. Additionally, the SOC is appreciable in the low-energy electronic properties of these two materials. The massless Dirac fermions of monolayer graphene primarily come from a honeycomb lattice with an underlying geometric symmetry. This yields a gapless semiconductor whose DOS vanishes at the Fermi level. Silicene and germanene are semiconductors and possess a direct band gap, in which the Dirac cones are distorted and separated by the significant SOCs. All the group-IV 2D systems have both valley and spin degeneracies. Central to the manipulation of energy bands is engineering an energy gap which yields a material for semiconductor applications. This serves to diversify tailor-made electronic properties which we could efficiently utilize.

As far as we are aware, layered silicene has been successfully synthesized on different substrates, such as Ag(111), Ag(110), CaF_2_, and CaSi_2_^4–12^. Experimental measurements have provided details for the structural, electronic, optical, and transport properties of silicene sheets^4–9^. It has been noted that the influence of the substrate on monolayer silicene cannot be neglected due to the strong hybridization between Si and the substrate. Fortunately, the strong interlayer coupling and weaker interaction with the substrate were reported in multilayer silicene^10–12^. That is, bilayer silicene can reduce the ambient effects from a substrate and enhance the stability of the heterostructure. Up to now, the stabilized AB-bt bilayer silicene has been successfully grown on CaF_2_ substrate^12^. The AB-bt and AB-bb (bottom-bottom) configurations are verified by high-angle annular dark field scanning transmission electron microscopy. The AB-bt stacking with the lowest ground state energy among bilayer silicene systems is herewith chosen for a detailed investigation of its optical properties. The theoretical predictions on crucial physical properties of this material are necessary and urgent as they will be helpful for further experimental investigation.

Examination of the fundamental physical properties of 2D materials, including their electronic, optical, transport, and Coulomb excitations, is very helpful in justifying their importance in the field of nanotechnology applications, such as the novel designs of nano-electronics, nano-optics, and energy storage^24–30^. The main characteristics of the electronic properties and optical spectra are reflected in the polarized and hyperspectral imaging for target identification, as well as in the ultrafast light-intensity modulations of space-laser transmission. In particular, unique optical excitations can be employed in the design of an electro-optic sensor system which is easy to transport and assemble. Additionally, the valley-, orbital-, and spin-dependent LLs under a magnetic field provide effective approaches for controlling angular momenta and spin projections of Fermion electrons. The spatial distribution of two electron spins when being transported by either spin-orbital coupling (SOC) or random impurity scattering constitutes a basis for modern spintronics and valleytronics. Such characteristics are taken into account in the design of next-generation ultrafast transistors for on-chip image processing in photo-detection. Novel phenomena in Coulomb excitations are utilized to design easily transportable, compact, low-power and reconfigurable devices in security and wideband optical communications.

An important component in our calculations is the method for diagonalizing the Hamiltonians of emergent layered materials. We have adapted the generalized tight-binding model and employed the dynamic Kubo formula from linear response theory in our numerical calculations. This method has certain advantages over other techniques which have been widely used by many researchers in the study of magnetic properties in condensed matter systems. Understandably, the huge magnetic Hamiltonian (e.g., more than 10^4^ × 10^4^ matrix for AB-bt bilayer silicene under *B*_0_ = 40 T) cannot be solved using Density Functional Theory. Moreover, the low-energy effective mass approximation usually ignores the interlayer hopping integrals of layered systems, leading to uncertainty in calculated critical results. For AB-bt bilayer silicene, this method is impossible to extend to include low-energy electronic states from the **K** and **T** points simultaneously. It is also noticed that the tight-binding method developed using the $\overrightarrow{{\rm{k}}}-$ scheme cannot provide reliable results for several important features of quantized LLs, such as the spatial distribution and localization behavior^31,32^. The above mentioned difficulties can be overcome within the tight-binding model used in this work for which the calculations are based on the sublattices in an enlarged unit cell in real space. This procedure is capable of including all critical ingredients simultaneously, including the single- or multi-orbital chemical bondings, SOCs, magnetic and electric fields, uniform or modulated external fields, intralayer and interlayer hopping integrals, arbitrary numbers of layers, various stacking configurations, planar or curved surfaces, and hybridized structures^33,34^. It can considerably diversify the electronic and optical properties in calculating the band structures, valley and spin degeneracies, energy gap or band overlap, Van Hove singularities of the DOS, LL crossings and anti-crossings, magneto-optical selection rules, and various absorption structures.

**Figure 1:**
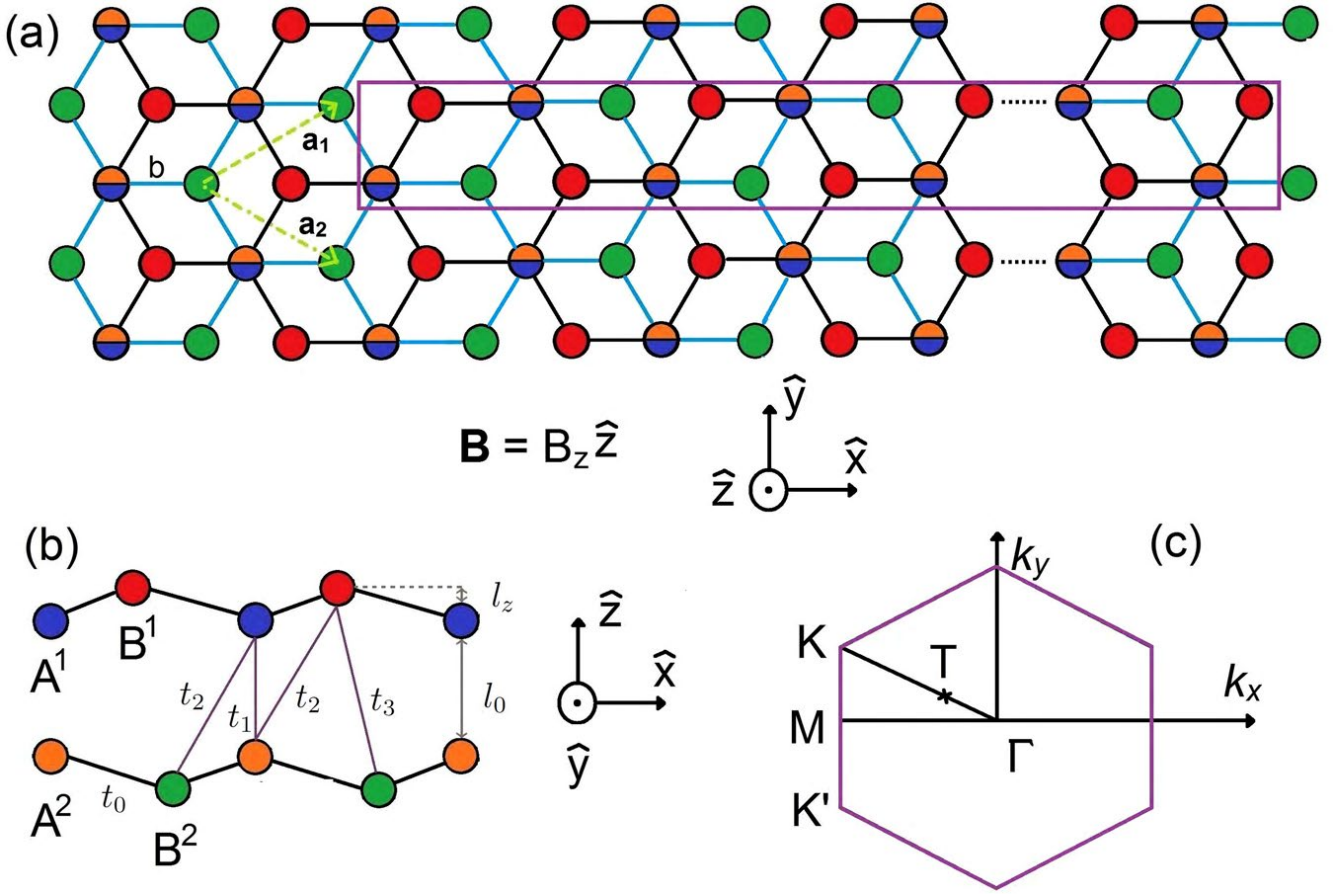
The geometric structure of AB-bt bilayer silicene with the top view (**a**) and side view (**b**). The enlarged unit cell under a uniform perpendicular magnetic field is marked by the purple rectangular in (**a**). The intra- and inter-layer atomic interactions are presented in (**b**). The first Brillouin zone with the highly symmetric **K** (**K′**) and **Γ** points and an extreme one, **T**, is shown in (**c**).

The atomic structure with intra- and inter-layer atomic interactions and buckling order are depicted in Fig. 1, for which the first Brillouin zone is the same as that of layered graphene. With the significant SOCs, an electric field can destroy the (*x*, *y*)-plane mirror symmetry and thus lead to valley- and spin-split states in the AB-bt configuration. These critical factors dominate the low-energy physical properties. It is well known that a uniform perpendicular magnetic field creates highly degenerate LLs by quantization of the neighboring electronic states. The well-behaved LLs possess symmetric/antisymmetric spatial distributions in a localized range and their energy spectra display band-induced field dependencies. There also exist some perturbed/undefined LLs with frequent anti-crossing behaviors. Specifically, the generalized tight-binding model can be used in conjunction with many-body and single-particle theories when the eigenstates are represented in terms of sublattice envelope functions. The unified models are appropriate for investigating a variety of properties including electronic properties, optical conductivities, quantum Hall effect, and Coulomb excitations.

## Method

The generalized tight-binding model used in our calculations includes all the critical factors simultaneously, the intra- and inter-layer atomic interactions, layer-dependent traditional and Bychkov-Rashba SOCs, and external electric and magnetic fields. Regarding the bottom-top configuration, the first and second layers present opposite buckled ordering, as shown in Fig. 1(b). Consequently, the significant vertical interlayer atomic interaction and SOCs dominate the low-lying electronic properties and absorption spectra.

The tight-binding Hamiltonian may be written as^27^

$$\begin{array}{lll}H & = & \sum _{m,l,\alpha }({\epsilon }_{m}^{l}+{U}_{m}^{l}){c}_{m\alpha }^{\dagger l}{c}_{m\alpha }^{l}+\sum _{m,j,\alpha ,l,l^{\prime} }{t}_{mj}^{ll^{\prime} }{c}_{m\alpha }^{\dagger l}{c}_{j\alpha }^{l^{\prime} }\\  &  & +\,\frac{i}{3\sqrt{3}}\sum _{\langle \langle m,j\rangle \rangle ,\alpha ,\beta ,l}{\lambda }_{l}^{SOC}{\gamma }_{l}{v}_{mj}{c}_{m\alpha }^{\dagger l}{\sigma }_{\alpha \beta }^{z}{c}_{j\beta }^{l}\\  &  & -\,\frac{2i}{3}\sum _{\langle \langle m,j\rangle \rangle ,\alpha ,\beta ,l}{\lambda }_{l}^{R}{\gamma }_{l}{u}_{mj}{c}_{m\alpha }^{\dagger l}{(\overrightarrow{\sigma }\times {\hat{d}}_{mj})}_{\alpha \beta }^{z}{c}_{j\beta }^{l}.\end{array}$$

In this notation, ${c}_{m\alpha }^{l}$ (${c}_{m\alpha }^{\dagger l}$) is the anihilation (creation) operator, which destroys (creates) an electronic state at the *m*-th site of the *l*-th layer with spin polarization *α*. The site energy, ${\varepsilon }_{m}^{l}({A}^{l},{B}^{l})$, arising from the chemical environment difference between the *A*^*l*^ and *B*^*l*^ sublattices, are defined as ${\varepsilon }_{m}^{l}({A}^{l})$ = 0 and ${\varepsilon }_{m}^{l}({B}^{l})=-\,0.12$ eV. The height-induced Coulomb potential energy, ${U}_{m}^{l}({A}^{l},{B}^{l})$, comes from the applied electric field. The intra- and inter-layer hopping integrals, ${t}_{mj}^{l{l}^{\text{'}}}$, are related to the neighboring atomic interactions. The former (*t*_0_ = 1.13 eV) and the latter (*t*_1_ = −2.2 eV, *t*_2_ = 0.1 eV; *t*_3_ = 0.54 eV) are clearly illustrated in Fig. 1(b). AB-bt bilayer silicene possesses significant layer-dependent SOCs (the third and fourth terms), mainly owing to the very strong orbital hybridizations induced by the large inter-layer vertical hopping integral. They are optimized (${\lambda }_{1}^{SOC}\,=\,0.06\,$ eV, ${\lambda }_{2}^{SOC}\,=\,0.046\,$ eV, ${\lambda }_{1}^{R}=-\,0.054\,$ eV; ${\lambda }_{2}^{R}=-\,0.043\,$ eV) in order to reproduce the low-lying energy bands calculated by the first-principles method^34–36^.

If AB-bt bilayer silicene is subjected to a uniform perpendicular magnetic field, the Hamiltonian becomes a huge Hermitian matrix. The enlarged unit cell due the vector potential (details in ref.^34^) is demonstrated in Fig. 1(a), e.g., 10^4^ silicon atoms under *B*_*z*_ = 40 T. For such a complex system, solving the eigenvalues and eigenstates is difficult. We need to employ the band-like method and the spatial localizations of the magnetic wavefunctions to efficiently solve the LL eigenvalues and eigenfunctions. Moreover, based on the *B*_*z*_-dependent evolution of LL energies, we can predict the main characteristics of LLs in a laboratory-produced field from those at higher field. Each LL wavefunction, with the quantum number n, is given by

1 $${\rm{\Psi }}(n,{\bf{k}})=\sum _{l=\mathrm{1,2}}\sum _{m=1}^{{R}_{B}}\sum _{\alpha ,\beta }[{A}_{\alpha ,\beta }^{l,m}(n,{\bf{k}})|{\psi }_{\alpha ,\beta }^{l,m}(A)\rangle +{B}_{\alpha ,\beta }^{l,m}(n,{\bf{k}})|{\psi }_{\alpha ,\beta }^{l,m}(B)\rangle ]\mathrm{.}$$

We have ${\psi }_{\alpha ,\beta }^{l,m}$ as the tight-binding function and ${A}_{\alpha ,\beta }^{l,m}(n,{\bf{k}})$ (${B}_{\alpha ,\beta }^{l,m}(n,{\bf{k}})$) denotes the amplitude of the spatial distribution. They are strongly dependent on the lattice sites.

When AB-bt bilayer silicene is present in an electromagnetic field, electrons are vertically excited from occupied states to unoccupied ones with the same wave vectors. The generalized tight-binding model combined with the dynamic Kubo formula is suitable for thoroughly exploring the optical excitations in the presence/absence of external fields. The zero-temperature spectral function related to the optical conductivity (*A*(*ω*) ∝ *ωσ*(*ω*)) is

2 $$A(\omega )\propto \sum _{c,v,m,m^{\prime} }{\int }_{1stBZ}\frac{d{\bf{k}}}{{\mathrm{(2}\pi )}^{2}}{|\langle {{\rm{\Psi }}}^{c}({\bf{k}},m^{\prime} )|\frac{\hat{{\bf{E}}}\cdot P}{{m}_{e}}|{{\rm{\Psi }}}^{v}({\bf{k}},m)\rangle |}^{2}\times Im[\frac{1}{{E}^{c}({\bf{k}},m^{\prime} )-{E}^{v}({\bf{k}},m)-\omega -i\Gamma }]\mathrm{.}$$

The absorption function is associated with the velocity matrix element (the first term in Eq. (3)) and the joint density of states (JDOS; the second term). The former, which is evaluated from the gradient approximation, as successfully utilized in layered graphene systems^36^, can determine the existence of vertical excitations/inter-LL transitions. That is, this dipole moment dominates the magneto-optical selection rules from the symmetries of the spatial distributions in the initial and final states. The latter reveals the van Hove singularities as the special absorption structures. The generalized tight-binding model, combined with the Kubo formula, is useful for the thorough investigation of essential physical properties in many 2D materials, such as graphene^33^, silicene^34^, tinene^17^, phosphorene^20^, bismuthene^23^, and others.

## Results and Discussion

**Figure 2:**
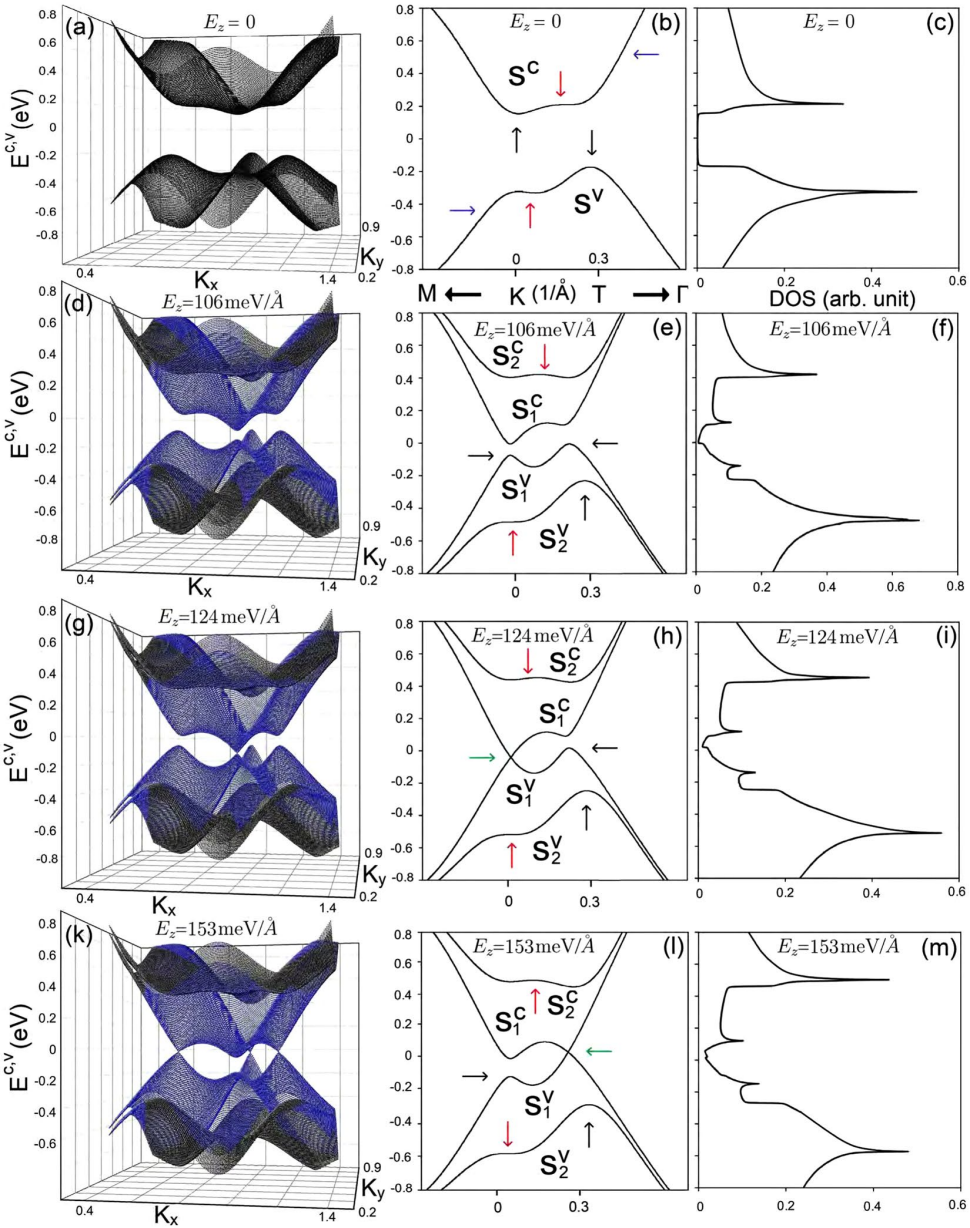
The 3D band structures, 2D energy bands along the high symmetry points and density of states (**a**–**c**) for *E*_*z*_ = 0, (**d**–**f**) *E*_*z*_ = 106 meV/Å, (**g**–**i**) *E*_*z*_ = 124 meV/Å, and (**k**–**m**) *E*_*z*_ = 153 meV/Å.

AB-bt bilayer silicene exhibits a unusual electronic structure, being characterized by the silicon 3*p*_*z*_ orbitals for the low-lying bands, as shown in Fig. 2. It covers the extremal band-edge states (black arrows), the partially flat energy dispersions along the specific direction (red arrows), the constant-energy loops (blue arrows), and the linear Dirac-cone structures (green arrows). The second and third types could be regarded as 1D parabolic dispersions, revealing similar peak structures in the DOS, which we discuss below. The zero-field energy band is in agreement with that obtained from the first-principles method^31^. Interestingly, the valence and conduction bands are sensitive to an external electric field. They are split into two pairs of spin-up- and spin-down-dominated energy subbands (${S}_{\mathrm{1,2}}^{c}$, ${S}_{\mathrm{1,2}}^{v}$), as clearly indicated in Fig. 2(b,e,h,k). In the presence of *E*_*z*_, spin degeneracy is absent due to the elimination of mirror symmetry. With increasing electric field strength, the low-lying pair of energy subbands (${S}_{1}^{c}$, ${S}_{1}^{v}$) gradually approach each other until the band gap is completely closed at the first critical field of *E*_*z*_ ≈ 106 meV/Å, as is evident in Fig. 2(d–e), where the highest occupied valence state and the lowest unoccupied one have different wave vectors. On the other hand, the outer pair of (${S}_{2}^{c}$, ${S}_{2}^{v}$) energy subbands monotonically moves away from the Fermi level. Beyond the critical electric field, the valence and conduction bands exhibit a slight overlap, and the linear Dirac cone structures are formed at the **K** (**K**′) point for the second critical electric field of *E*_*z*_ = 124 meV/Å (Fig. 2(g,h)) and at the **T** point for the third one of *E*_*z*_ = 153 meV/Å (2(k) − 2(l))). The Dirac points change from the occupied valence states into the unoccupied conduction states with the further increase of *E*_*z*_.

The density-of-states directly reflects the main features of the unusual energy bands, as shown in Fig. 2(c,f,i and m). At zero field, the large band gap is clearly revealed in the zero-field DOS in 2 (c). The low-frequency DOS presents prominent asymmetric peaks, respectively, corresponding to the extremal band-edge states and the partially flat bands along the specific direction in the energy-wave-vector space. In the presence of an electric field, the splitting of energy bands gives rise to more shoulder-like and peak structures, as illustrated in Fig. 2(f,i and m). Additionally, the *E*_*z*_-induced constant-energy loops and Dirac cones, respectively, create the asymmetric peaks and valley-like structures, in which the latter appear near the Fermi level. The *E*_*z*_-dependent energy gap and three kinds of Van Hove singularities could be directly identified from experimental measurements using scanning tunneling spectroscopy (STS)^37,38^. Also, STS is the most powerful tool relating the measured tunneling current to the DOS.

**Figure 3:**
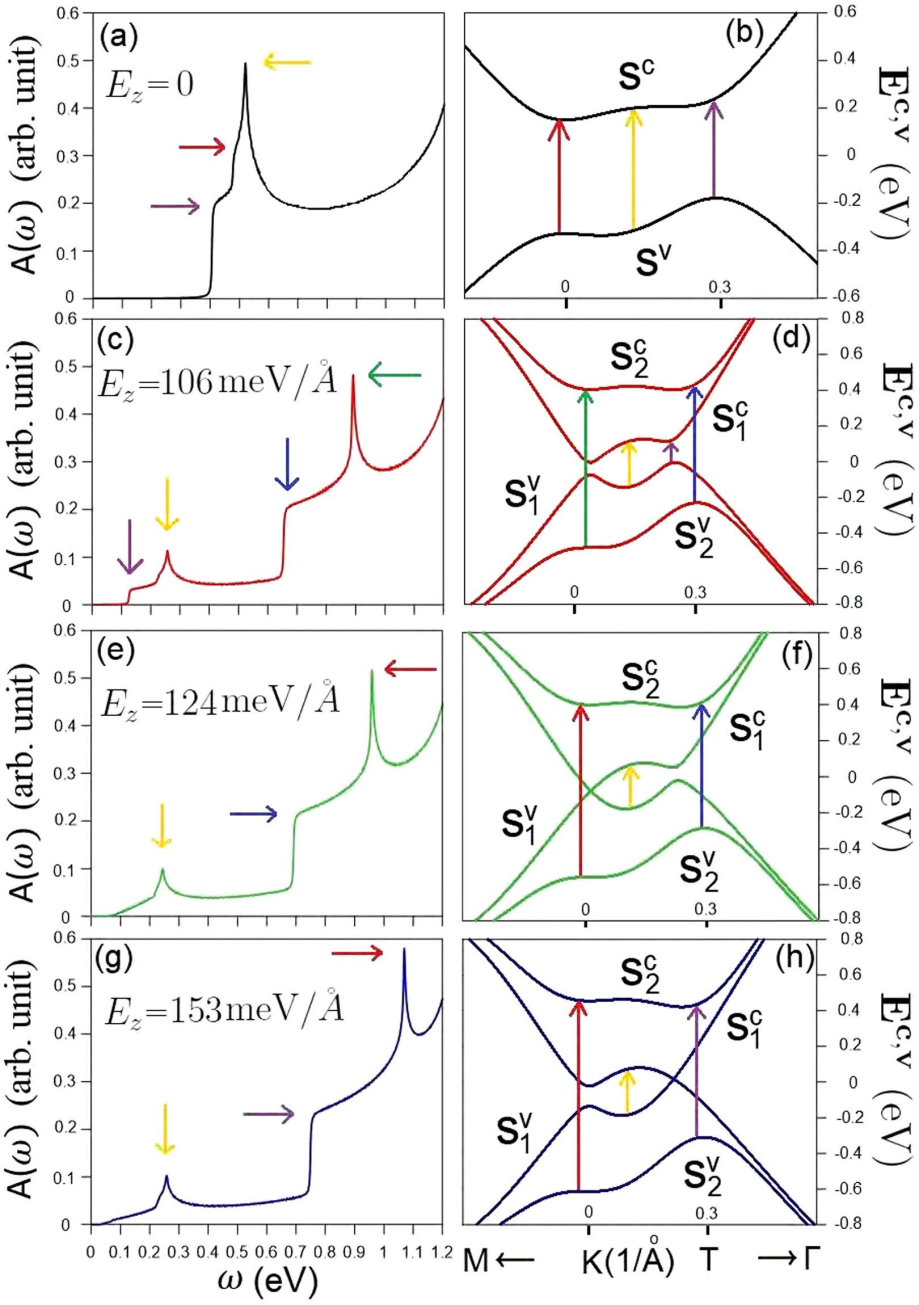
The optical-absorption spectra of bilayer silicene in the absence of *E*_*z*_ (**a**) and the corresponding optical channels (**b**). Similar figures are shown for (**c**,**d**) *E*_*z*_ = 106 meV/Å, (**e**,**f**) *E*_*z*_ = 124 meV/Å, and (**g**,**h**) *E*_*z*_ = 153 meV/Å.

The optical transitions of AB-bt bilayer silicene present a feature-rich absorption spectra, in sharp contrast with those of the monolayer system^5^. There are three special structures in optical excitations, as clearly shown when *E*_*z*_ = 0 by the black curve in Fig. 3(a). The first two belong to the shoulder structures, in which the first and second ones arise from the band-edge states at the **K** and **T** points (purple and red arrows in Fig. 3 and (b)), respectively. The third structure, the antisymmetric peak (yellow arrow), is due to the weak energy dispersion close to the **K** point. All of them are dominated by Van Hove singularities in the JDOS under vertical excitations. An electric field makes the low-lying optical excitations become more complicated in the presence of the spin-split energy bands. There are two absorption regions, since vertical transitions are allowed only for the same pair of valence and conduction bands (${S}_{1}^{v}\to {S}_{1}^{c}$ and ${S}_{2}^{v}\to {S}_{2}^{c}$). The lower- and higher-frequency absorption regions have similar structures, i.e., the shoulder and peak, when the electric filed is not too strong (e.g., red curve in Fig. 3(c) for *E*_*z*_ ≤ 106 meV/Å). The former and the latter come from the optical transitions of the band-edge states near the **T** point and the electronic states from the flat bands near the **K** (Fig. 3(d)), respectively. The lowest-frequency threshold shoulder disappears for the higher electric field, e.g, *E*_*z*_ = 124 meV/Å and *E*_*z*_ = 153 meV/Å, because the band-edge states of the first valence/conduction band are also unoccupied/occupied near the **T**/**K** point (Fig. 3(e–h)). The above electric-field-enriched optical structures could be examined by infrared reflection spectroscopy and absorption spectroscopy^39,40^.

**Figure 4:**
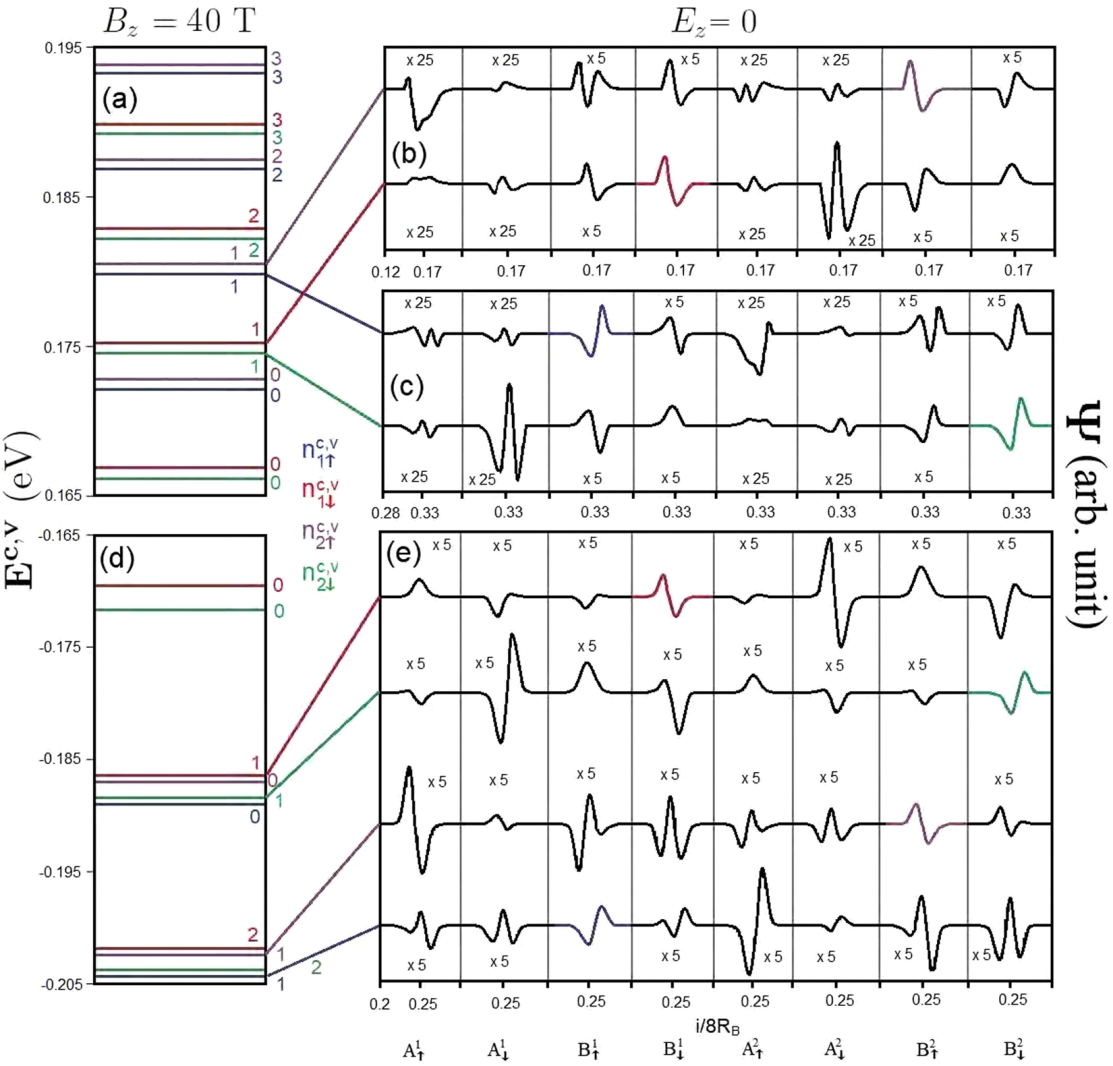
The (**a**) LL spectrum and (**b**,**c**) conduction LL wavefunctions on eight spin-split distinct sublattices for *B*_*z*_ = 40 T in the absence of electric field. Similar plots for the valence LL spectrum (**d** and **e**) wavefunctions are also presented.

This material shows rich magneto-electronic properties, being thoroughly different from bilayer graphene. The buckled structure, complex inter-layer atomic interactions, and significant SOCs remarkably enrich the main features of LLs. The low-lying conduction and valence LLs are quantized from the electronic states near the **K** and **T** points, respectively. They are doubly degenerate under the interplay of nonequivalent sublattices and SOCs, while there exist the eight-fold degeneracy in bilayer graphene. The conduction LL wavefunctions are centered at 1/6 (4/6) and 2/6 (5/6) of the expanded unit cell while the valence ones are localized at 1/4 (3/4) center. Such wavefunctions are the well-behaved spatial distributions characterized by the 3*p*_*z*_− and spin-dependent sub-envelope functions on the eight sublattices, as demonstrated in Fig. 4. Their quantum numbers are defined by the number of zero points of the spatial probability distributions in the dominated sublattices. Furthermore, they are very useful in understanding the magneto-optical selection rules of the inter-LL transitions. In principle, LLs can be classified as four distinct subgroups (${n}_{\uparrow 1}^{c}$, ${n}_{\downarrow 1}^{c}$, ${n}_{\uparrow 2}^{c}$ and ${n}_{\downarrow 2}^{c}$) based on the sublattice- and spin-dominated wavefunctions (blue, red, purple, and green lines in Fig. 4(a,d)). Four LL subgroups possess the usual orderings of state energy and energy spacing; that is, such properties, respectively, grow and decline with the increase/decrease of *E*^*c*^/*E*^*v*^. The LL energy splitting is induced by both SOCs and stacking configuration/interlayer atomic interactions, in which the former is much larger than the later. The split energy is strongly dependent on the magnetic field strength (discussed later).

**Figure 5:**
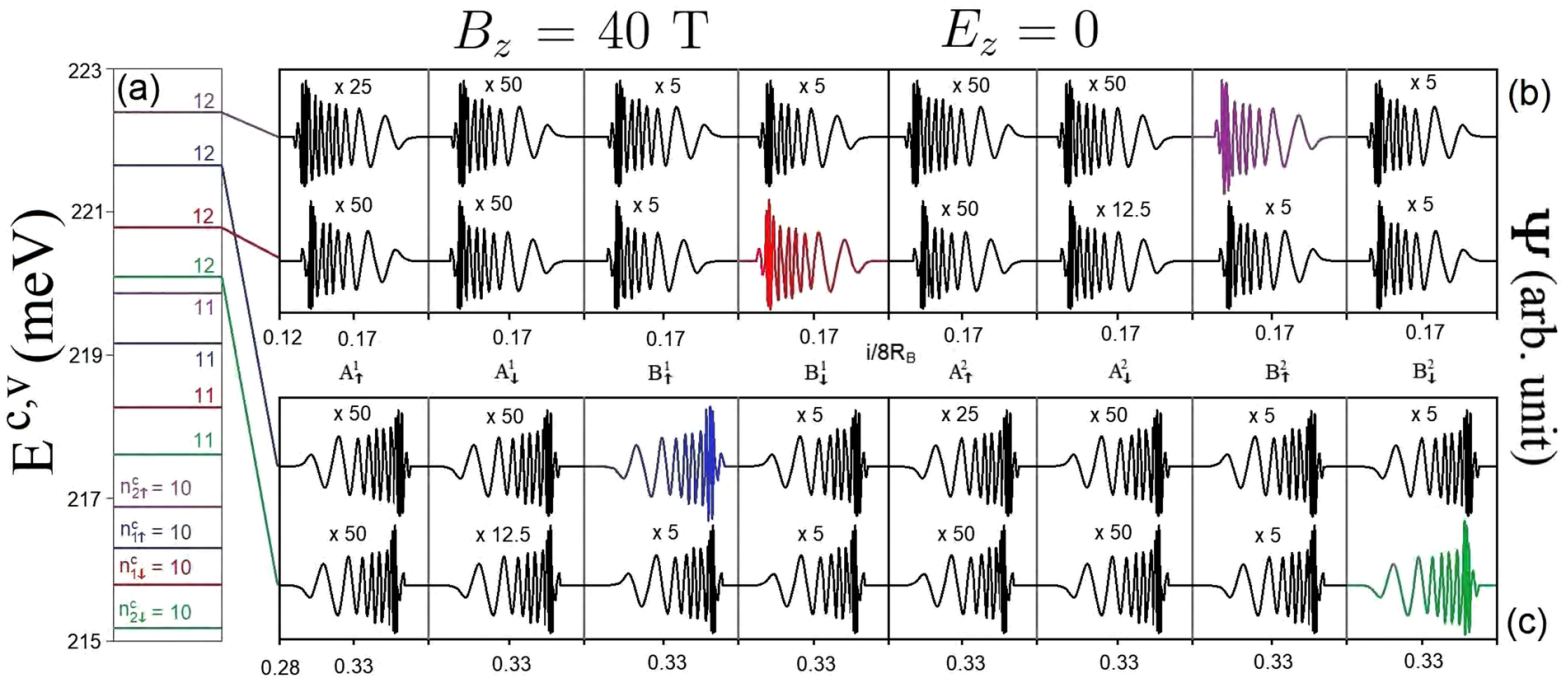
The (**a**) higher conduction LL spectrum and (**b**,**c**) LL wavefunctions on eight distinct sublattices for *B*_*z*_ = 40 T in the absence of electric field.

Since the low-lying valence and conduction LLs have different localization centers, the vertical magneto-optical transitions between them are forbidden. The higher conduction and deeper valence LLs, with many oscillation modes, are critical in understanding the magneto-optical excitations; therefore, they deserve closer observation. They are, respectively, quantized from the electronic states near **T** and **K** valleys, corresponding to the shoulder-like energy bands (Fig. 2(b)). This is thoroughly different from the magnetic quantization in bilayer graphene systems^3^. The energy spectrum and spatial distributions of the higher conduction LLs are clearly illustrated in Fig. 5(a–c)). They clearly exhibit the non-symmetric and non-well-behaved spatial distributions; that is, they belong to the perturbed LLs with main and side modes^3^. For the higher conduction/deeper valence LLs, their contribution widths are wide and the effective width covers two localization centers of (1/6, 1/4) & (2/6, 1/4). As a result, the low-lying conduction/valence and the deeper valence/higher conduction LLs will have a significant overlap in the spatial distributions, becoming very important in the magneto-optical threshold excitation.

**Figure 6:**
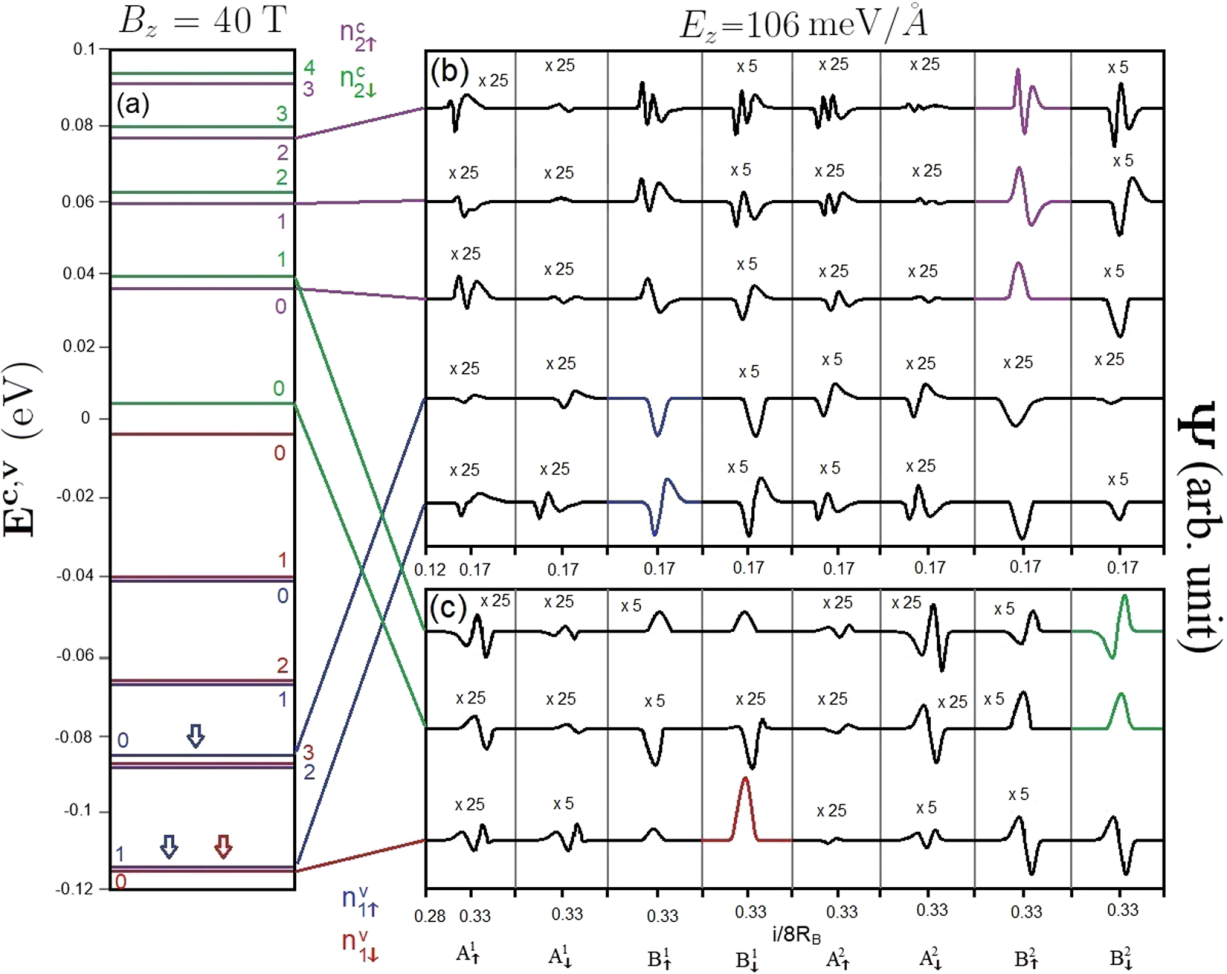
The (**a**) LL spectrum and (**b**–**d**) LL wavefunctions on eight distinct sublattices for *B*_*z*_ = 40 T under *E*_*z*_ = 106 meV/Å.

The magneto-electronic properties can be greatly diversified by an external electric field. For the zero-gap band structure under a critical electric field, *E*_*z*_ = 106 meV/Å, the magnetic quantization is initiated from the valence and conduction states near the **K** and **T** valleys (Fig. 6). There exist the low-lying valence and conduction LLs with the same localization center simultaneously, instead of only conduction or valence ones as for *E*_*z*_ = 0. In particular, the well-behaved valence LLs at 1/6 and 2/6 centers (the conduction ones at 1/4 center) come into existence, as marked by the arrows in Fig. 6(a). Such LLs come from the rather pronounced oscillating band structure in the presence of an electric field (Fig. 2(e)). The LL energy spectrum and spatial distributions for this critical electric field are clearly illustrated in Fig. 6(a–c). Four subgroups of LLs do not appear together, but are separated into two lower- and higher/deeper-energy ones. This means that, the splitting related to the non-equivalence of sublattices is greatly enhanced by the electric field because of the distinct Coulomb site energies. We only focus on the former associated with the lower-frequency magneto-optical excitations. For each valley, the two subgroups of low-lying conduction and valence LLs correspond to (${n}_{\uparrow 2}^{c}$, ${n}_{\downarrow 2}^{c}$) and (${n}_{\uparrow 1}^{v}$, ${n}_{\downarrow 1}^{v}$), respectively. It should be noticed that they are dominated by the different sublattices. The vertical transitions, valence to conduction LLs, from the different valleys provide a major contribution to the magneto-optical spectra.

**Figure 7:**
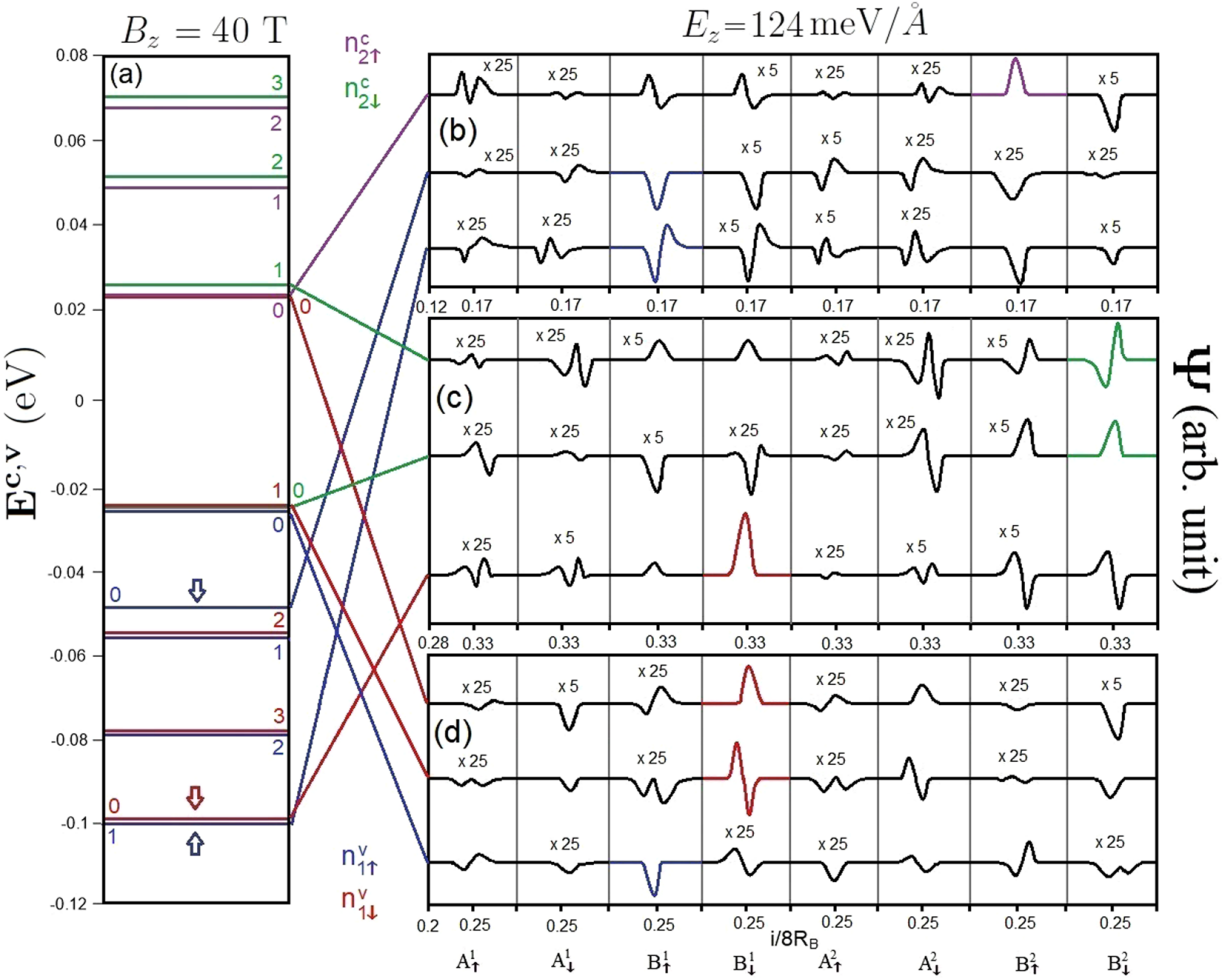
The (**a**) LL spectrum and (**b**–**d**) LL wavefunctions on eight distinct sublattices for *B*_*z*_ = 40 T under *E*_*z*_ = 124 meV/Å.

On the other hand, a perpendicular electric field may lead to the formation of Dirac cones at the **K** or **T** valleys, giving rise to special LL quantization. For the second critical electric field, *E*_*z*_ = 124 meV/Å, the conduction and valence LLs which are initiated from the **K** and **T** valleys, respectively, correspond to (${n}_{\downarrow 2}^{c}$, ${n}_{\uparrow 2}^{v}$) and (${n}_{\downarrow 1}^{c}$, ${n}_{\uparrow 1}^{v}$), in which all the LL wavefunctions are well-behaved in the spatial distribution, as clearly shown in Fig. 7. The former is similar to those from the linear Dirac cone^26^, their energies are characterized by ${E}_{\mathrm{2(1)}}^{c(v)}$ $\propto \sqrt{{n}_{\downarrow 2}^{c}({n}_{\uparrow 1}^{v})}$. A simple relation is absent for the latter. Specifically, the energy spacing of ${n}_{\downarrow 2}^{c}$ = 0 and ${n}_{\uparrow 1}^{v}$ = 0 is finite and gradually grows with the magnetic field strength, having a magnitude of 25 meV at *B*_*z*_ = 40 T. The opposite is true for the third critical electric field, *E*_*z*_ = 153 meV/Å. That is, the Dirac cone-like LLs are created by the electronic states near the **T** valley. The sharp contrast between the **K** and **T** valleys will be directly reflected in the magneto-optical excitations. Moreover, there exist certain important differences compared to monolayer graphene with a zero energy spacing between the *n*^*c*^ = 0 and *n*^*v*^ = 0 LLs, eight-fold degenerate LLs, and the same dominating sublattices for the valence and conduction LLs^33^.

**Figure 8:**
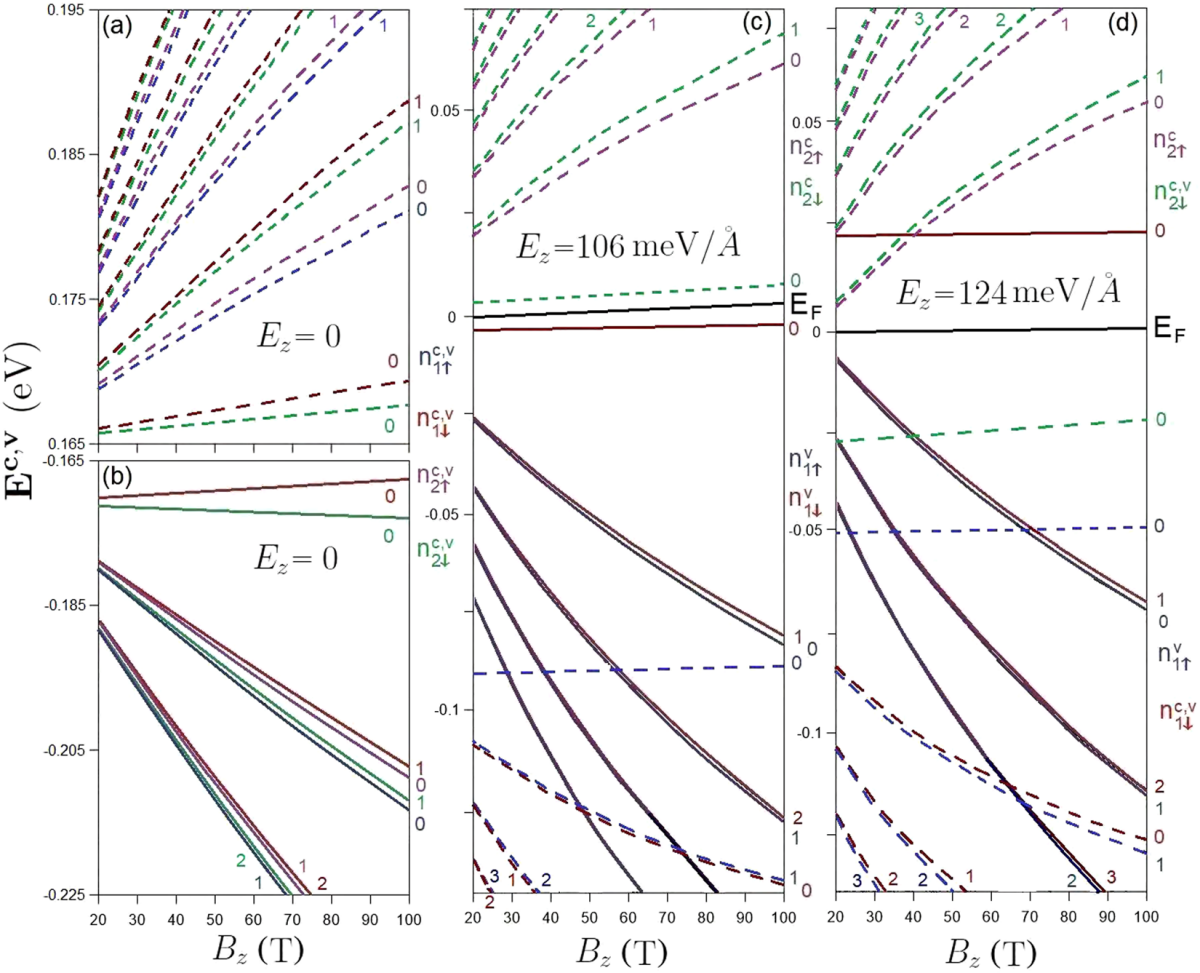
The *B*_*z*_-dependent LL energy spectra in the absence of electric field (**a**) and under (**b**) *E*_*z*_ = 106 meV/Å and (**c**) *E*_*z*_ = 124 meV/Å. The LLs near the *K* and *T* valleys are presented by the dashed and solid curves, respectively.

The *B*_*z*_-dependent LL energy spectrum is very useful for the comprehension of magnetic quantization. Without an electric field, the conduction/valence LL energies are generally increased/decreased with the growth of magnetic field strength, as clearly shown in Fig. 8(a,b) by the dashed and solid curves (initiated from the **K** and **T** valleys). As an exception, the initial valence ${n}_{\downarrow 1}^{v}$ = 0 LL energy slowly grows with *B*_*z*_. The energy gap, which is determined by the ${n}_{\downarrow 2}^{c}$ = 0 and ${n}_{\downarrow 1}^{v}$ = 0 LLs, remains almost the same (*E*_*g*_(*B*_*z*_) ≈ 340 meV). The valence and conduction LL energy spectrum is asymmetric about the Fermi level, mainly owing to the important interlayer hopping integrals and layer-dependent SOCs. Four subgroups of LLs behave similarly during the variation of *B*_*z*_, in which the low-lying spectrum presents an almost linear *B*_*z*_-dependence, except for the initial valence ${n}_{\downarrow 1}^{v}$ = 0 LL near the **T** valley. This reflects the low-lying parabolic dispersions near the **K** and **T** valleys (Fig. 2(b)).

It should be noticed that the onset energies of LL subgroups are strongly dependent on the strength of the electric field. The effects of composite fields are clearly illustrated by the *B*_*z*_-dependent LL energy spectra at the critical electric field strengths, as shown in Fig. 8(c,d). The asymmetry of the LL energy spectrum is enhanced under the first critical electric field, *E*_*z*_ = 106 meV/Å (Fig. 8(c)). There are four initial LLs of (${n}_{\uparrow 1}^{v}=0$ & ${n}_{\downarrow 2}^{c}=0$) from the **K** valley and (${n}_{\downarrow 1}^{v}=0$ & ${n}_{\uparrow 2}^{c}=0$) from the **T** valley which present the weak *B*_*z*_-dependence, as demonstrated in Fig. 8(c). Specifically, the above-mentioned ${n}_{\downarrow 2}^{c}$ = 0 and ${n}_{\downarrow 1}^{v}=0$ LLs determine the Fermi level, being the middle of the nearest occupied and unoccupied LLs. The energy gap between these LLs becomes very narrow and grows with the magnetic field. It almost vanishes at sufficiently low *B*_*z*_. According to numerical examinations, the energy spacing and magnetic field strength present a neither simple linear nor square-root relationship. In addition, there are only a few well-behaved conduction LLs near the **T** valley (${n}_{\uparrow 2}^{c}$ & ${n}_{\downarrow 2}^{c}$) (the ${n}_{\uparrow 2}^{c}$ = 0 LL is observed for *B*_*z*_ ≥ 20 T) and they are located at relatively high energy compared with those initiated from the **K** valley. LLs near the **K** valley will dominate the threshold magneto-optical excitations.

Under the second critical electric field of *E*_*z*_ = 124 meV/Å, the conduction and valence LL subgroups initiated from the **K** valley approach each other, as shown in Fig. 8(d). This directly reflects the linear and isotropic Dirac-cone structures near the **K** point (Fig. 2(h)). As for the LLs quantized from the **T** valley, only the valence ones come to exist near the Fermi level, in which the ${n}_{\downarrow 1}^{v}=0$ LL (the red solid curve) is higher than the few ${n}_{\downarrow 2}^{c}$ conduction LLs from the **K** valley. Regarding the **K** valley, the zero-quantum-number LLs of ${n}_{\downarrow 2}^{c}$ and ${n}_{\uparrow 1}^{v}$ reach the minimum energy spacing. Furthermore, the LL energies and the magnetic field strength possess a specific relationship of ${E}_{\mathrm{2(1)}}^{c(v)}\propto \sqrt{{B}_{z}}$, similar to that in monolayer graphene^26^. Generally, the initial LLs of (${n}_{\uparrow 1}^{v}=0$ & ${n}_{\downarrow 2}^{c}$ = 0) and ${n}_{\downarrow 1}^{v}=0$ from the **K** and **T** valleys, respectively, are hardly affected by the magnetic field strength. The Fermi level is determined by the (${n}_{\downarrow 2}^{c}=0$ & ${n}_{\downarrow 1}^{v}=0$) LLs at sufficiently high magnetic field strength (greater and approximately equal to 40 T) and (${n}_{\uparrow 2}^{c}=0$ & ${n}_{\downarrow 1}^{v}=1$) for smaller *B*_*z*_. However, the value of *E*_*F*_ is almost independent of *B*_*z*_. An electric field can alter certain LLs nearest to the Fermi level, leading to dramatic changes in the magneto-optical threshold channels. For example, the valence ${n}_{\downarrow 1}^{v}$ = 0 LL from the **T** valley and conduction ${n}_{\uparrow 2}^{c}$ = 0 LL from the **K** valley become unoccupied and occupied, as illustrated in Fig. 8(d). The above-mentioned features are also revealed in the *B*_*z*_-dependent LL energy spectra for the third critical field of *E*_*z*_ = 153 meV/Å by means of the interchange of the **K** and **T** valleys. In general, an electric field creates more low-lying well-behaved conduction and valence LLs and thus is expected to induce very complicated magneto-optical absorption spectra.

**Figure 9:**
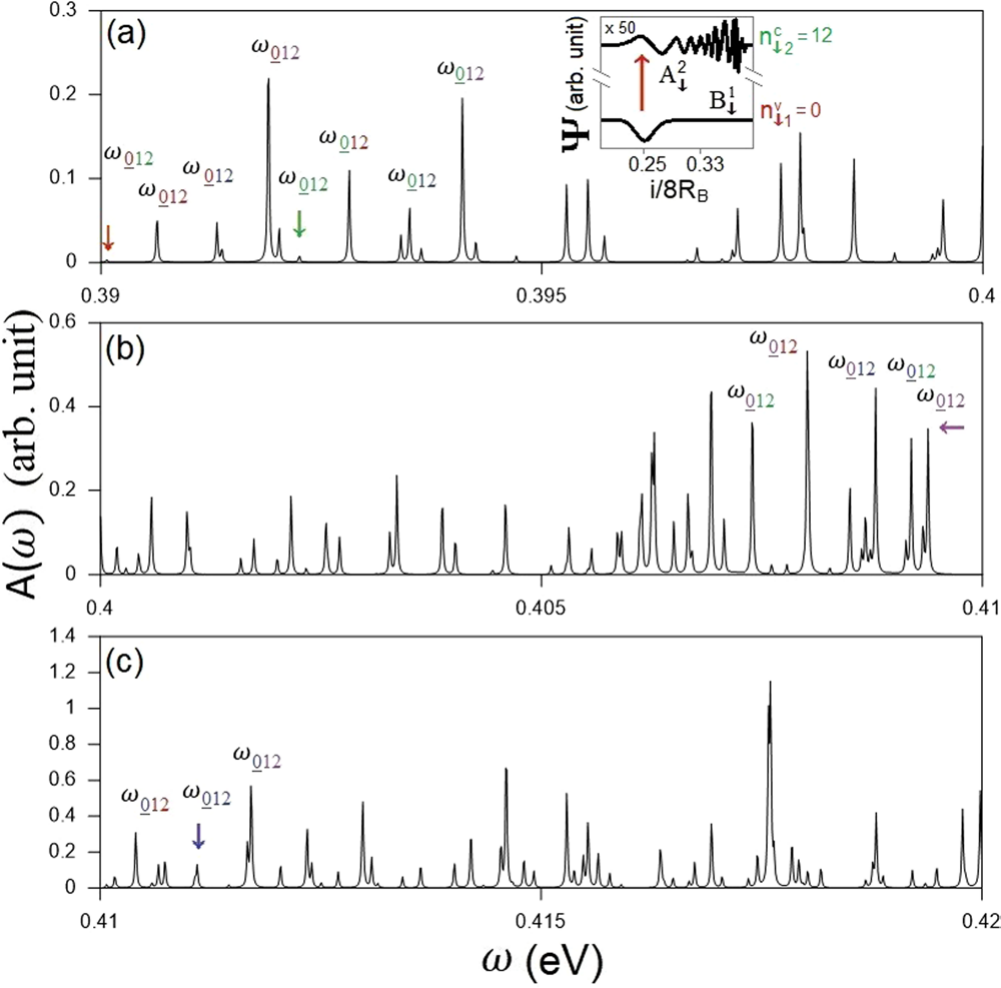
The spectral intensities of AB-bt bilayer silicene at *B*_*z*_ = 40 T in the absence of electric field.

AB-bt bilayer silicene presents the feature-rich magneto-absorption spectra, reflecting unusual LLs and band structures. The vertical transitions among four subgroups of LLs lead to many single, double and twin delta-function-like peaks with non-uniform intensities, as shown in Fig. 9(a–c). There are 4 × 4 categories of inter-LL optical transitions, covering 4 intra-subgroup (part of peaks are marked by the arrows with distinct colors) and 12 inter-subgroup ones. According to the magneto-optical absorption functions^33^, the inter-LL transition is available whenever the initial and final states related to the two sublattices in the large (*t*_0_, *t*_1_) hopping integrals possess the same quantization mode. As a result of an indirect energy gap, in each category, absorption peaks correspond to the optical transitions associated with the multi-mode LLs in the absence of a specific selection rule. This results in a lot of magneto-absorption peaks within a very narrow-range frequency of ∼30 meV, never observed in other condensed-matter systems^33,39–49^. For a LL with sufficiently large quantum number, the extended oscillation wavefunctions localized at (1/4)/(1/6 and 2/6) along the x-axis will overlap with that of another LL at the neighboring localization centers of (1/6 and 2/6)/(1/4). For example, the spatial distributions of the ${n}_{1\downarrow }^{v}=0$ and ${n}_{1\downarrow }^{c}=12$ LLs are illustrated in the inset of Fig. 9(a). The former and the latter are localized at 1/4 and 1/6 centers which are very close to each other, leading to an obvious overlapping phenomenon between them. This enables vertical optical transitions between the initial- and final-state LLs near the **T** (**K**) valley, in which the magneto-absorption peaks have different intensities and frequencies, e.g., the 16 absorption structures due to the (${n}_{1\downarrow }^{v}\,=\,0$, ${n}_{1\uparrow }^{v}\,=\,0$, ${n}_{2\uparrow }^{v}\,=\,0$, ${n}_{2\downarrow }^{v}\,=\,0$) and (${n}_{1\downarrow }^{c}=12$, ${n}_{1\uparrow }^{c}=12$, ${n}_{2\uparrow }^{c}=12$, ${n}_{2\downarrow }^{c}=12$) LLs. This very special magneto-optical property is absent in other well-known 2D systems, e.g., graphene^26^ and phosphorene^20^. The threshold absorption peak, the optical gap, belongs to the intra-subgroup ${n}_{1\downarrow }^{v}=0$ → ${n}_{1\downarrow }^{c}=12$ transition (red arrow in Fig. 9(a)). Its frequency is expected to be dependent on both magnetic and electric fields. The optical gap (≈0.39 eV for *B*_*z*_ = 40 T) is larger than the energy gap (≈0.3 eV), since the vertical transitions between the low-lying conduction and valence LLs at different centers are forbidden. In addition, there also exist 16 excitation categories related to the initial conduction LLs from the **K** valley, such as, those associated with the (${n}_{1\downarrow }^{v}=12$, ${n}_{1\uparrow }^{v}=12$, ${n}_{2\uparrow }^{v}=12$, ${n}_{2\downarrow }^{v}=12$) and (${n}_{1\downarrow }^{c}=0$, ${n}_{1\uparrow }^{c}=0$, ${n}_{2\uparrow }^{c}=0$, ${n}_{2\downarrow }^{c}=0$) LLs. However, they do not contribute to the threshold magneto-optical excitation.

**Figure 10:**
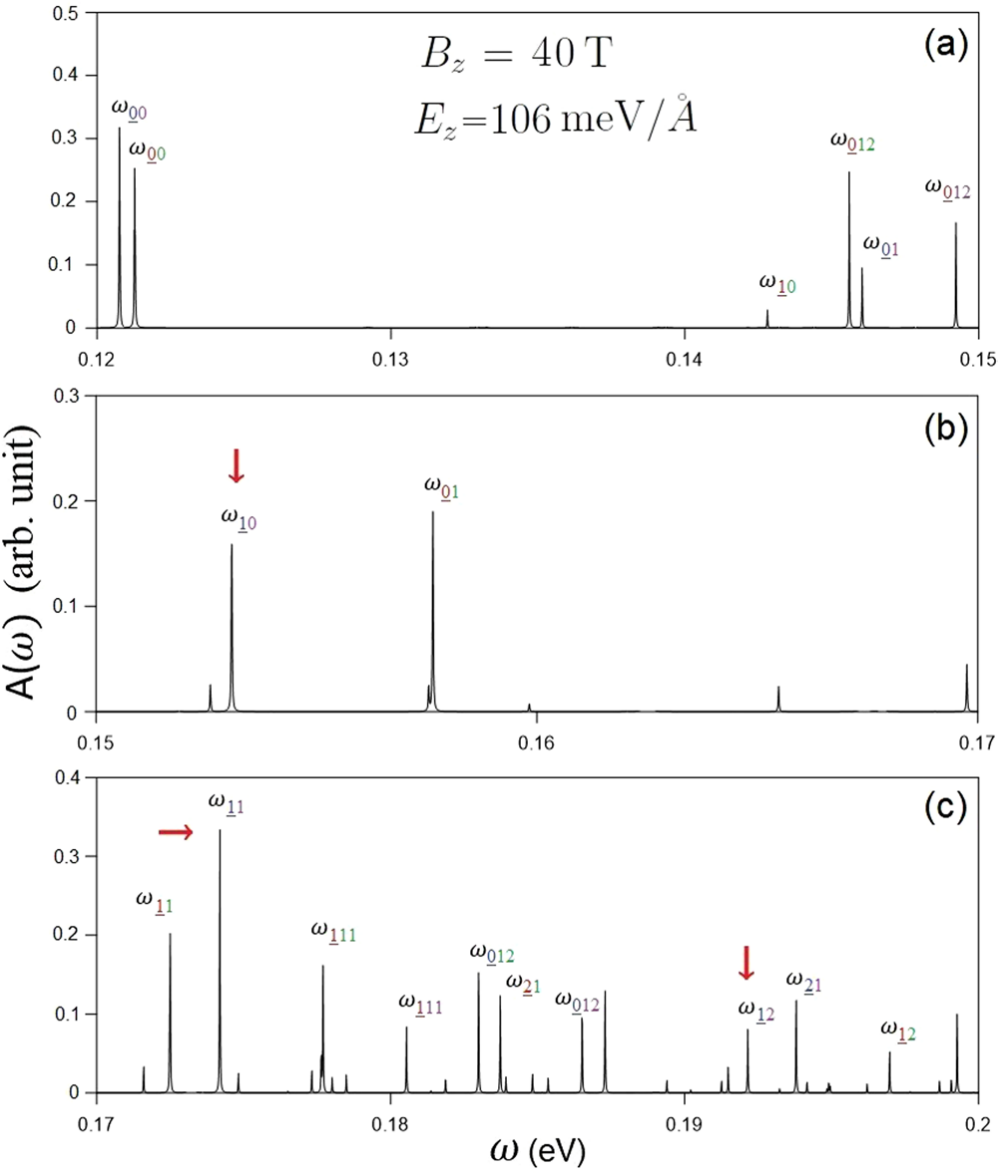
The spectral intensities of AB-bt bilayer silicene for *B*_*z*_ = 40 T under *E*_*z*_ = 106 meV/Å.

The magneto-optical spectra are diversified under the interplay of electric field, magnetic field, significant SOCs and interlayer atomic interactions. An applied electric field can create the inter-LL optical transitions at lower frequency, and they satisfy specific selection rules. There exist optical excitations related to LLs from the same localization center (type I) and neighboring ones (1/4 and 1/6 (2/6) centers) (type II). The type-I absorption peaks are associated with the *E*_*z*_-induced new low-lying well-behaved valence LLs at the **K** valley. They are only characterized by LLs with the same spin configuration (${n}_{\uparrow 1}^{v}$ → ${n}_{\uparrow 2}^{c}$ and ${n}_{\downarrow 1}^{v}$ → ${n}_{\downarrow 2}^{c}$). The threshold peak, which is determined by the ${n}_{\uparrow 1}^{v}=0$ → ${n}_{\uparrow 2}^{c}=0$ transition, is located at much lower frequency (*ω*_*th*_ ≈ 120 meV) compared with that at zero electric field. It should be noticed that, type-I magneto-optical excitations obey the optical selection rules of Δ*n* = 0 and ±1, as demonstrated in Fig. 10 for the first critical electric field (*E*_*z*_ = 106 meV/Å) under *B*_*z*_ = 40 T. This is because each LL possesses a main mode and a few side modes, referring to Fig. 6. For example, the ${n}_{\uparrow 1}^{v}$ = 1 LL contains a main mode of 1 on the dominating ${B}_{\uparrow }^{1}$ sublattice and the side modes of 0 and 2 on the other sublattices (Fig. 6(b)). As a result, there are available inter-LL optical transitions of ${n}_{\uparrow 1}^{v}=1$ → ${n}_{\uparrow 2}^{c}=0$, ${n}_{\uparrow 1}^{v}=1$ → ${n}_{\uparrow 2}^{c}=1$, and ${n}_{\uparrow 1}^{v}=1$ → ${n}_{\uparrow 2}^{c}=2$ = 2, as marked by the red arrows in Fig. 10(b,c). As for type-II absorption peaks, there are excitation channels between n = 0 and 12 LLs, similarly to those in the absence of an electric field; that is, they are associated with the low-lying well-behave valence LLs from the **T** valley. The above-mentioned absorption peaks, including both type-I and type-II ones, belong to 4 excitation categories, but not 16 ones as in the absence of *E*_*z*_. They cover the ${n}_{\uparrow 1}^{v}$ → ${n}_{\uparrow 2}^{c}$, ${n}_{\uparrow 1}^{v}$ → ${n}_{\downarrow 2}^{c}$, ${n}_{\downarrow 1}^{v}$ → ${n}_{\uparrow 2}^{c}$, and ${n}_{\downarrow 1}^{v}$ → ${n}_{\downarrow 2}^{c}$ magneto-optical transitions.

**Figure 11:**
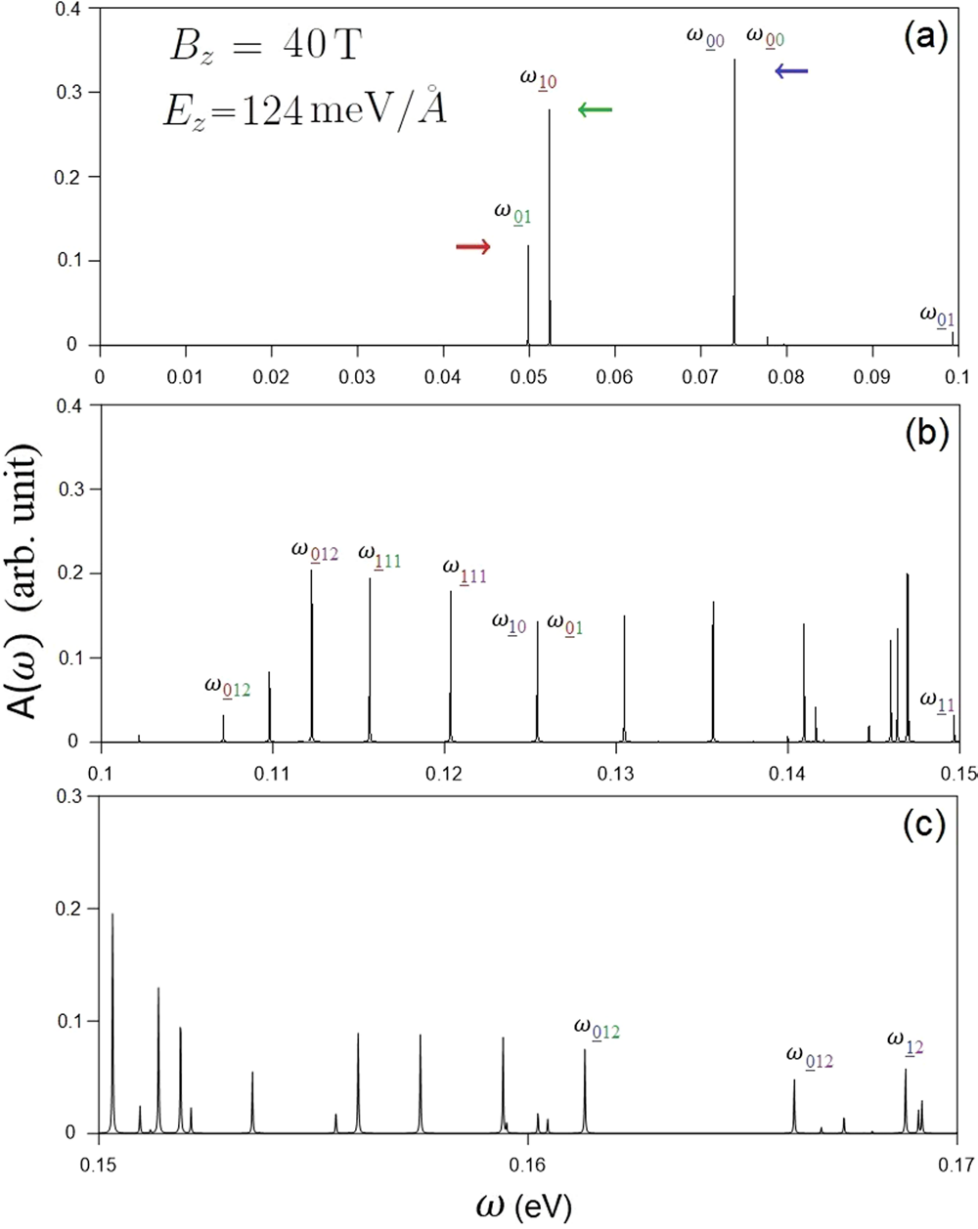
The spectral intensities of AB-bt bilayer silicene for *B*_*z*_ = 40 T under *E*_*z*_ = 124 meV/Å.

It is worth considering the magneto-optical spectra at the second critical electric field (*E*_*z*_ = 124 meV/Å) when a Dirac cone is formed at the **K** valley. Both type-I and type-II magneto-optical excitations come to exist in the absorption spectra. The former satisfy the optical selection rules of Δ*n* = 0 and ±1 while the later do not. Especially, the formation of a Dirac cone induces extraordinary phenomena. Since the Dirac cone lies below the Fermi level (Fig. 2(h)), the occupation of some LLs near the Dirac point is altered, which directly affects the threshold magneto-optical excitation (Fig. 11). The intra-subgroup excitation channels of ${n}_{\downarrow 2}^{c}=0$ → ${n}_{\downarrow 2}^{c}=1$ (red arrow) and ${n}_{\downarrow 1}^{v}=1$ → ${n}_{\downarrow 1}^{v}=0$ (green arrow) comes into existence because the conduction ${n}_{\downarrow 2}^{c}=0$ LL near the **K** valley and ${n}_{\downarrow 1}^{v}=0$ one near the **T** valley become occupied and unoccupied, respectively. The threshold absorption peak, which is determined by the former, is present at rather low frequency (*ω*_*th*_ ∼ 50 meV). Furthermore, there exists a double peak due to the two peaks of (${n}_{\uparrow 1}^{v}=0$ → ${n}_{\uparrow 2}^{c}=0$ and ${n}_{\downarrow 1}^{v}=0$ → ${n}_{\downarrow 2}^{c}=0$) merging together in the absorption spectrum, as indicated by the blue arrow in Fig. 11(a). The crossing behavior between LLs is responsible for this double peak. In general, type-I magneto-optical excitations are mainly revealed near the **K** valley, except for ${n}_{\downarrow 1}^{v}=1$ → ${n}_{\downarrow 1}^{v}=0$ (green arrow) near the **T** valley. Similar magneto-optical properties can also be observed for the third critical electric field (*E*_*z*_ = 153 meV/Å) when the Dirac cone at the **T** valley is located above the Fermi level.

**Figure 12:**
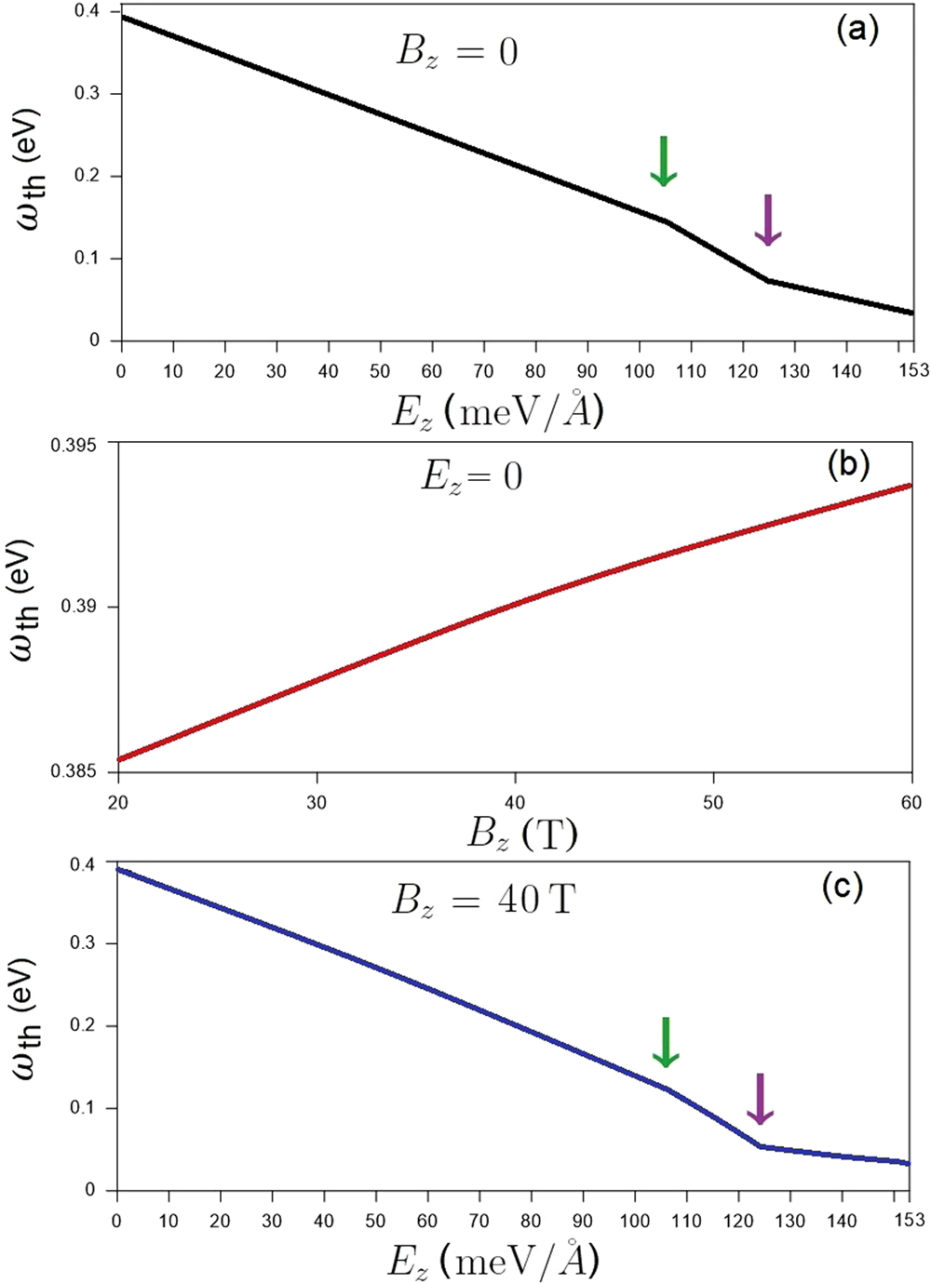
The (**a**) *B*_*z*_-dependent threshold frequencies at zero electric field and (**b**) *E*_*z*_-dependent threshold frequencies for *B*_*z*_ = 40 T.

The optical gap strongly depends on the strength of the electric and magnetic fields, as demonstrated in Fig. 12(a–c). With the increase of the electric field up to the first critical field (*E*_*z*_ = 106 meV/Å), the threshold frequency, which is determined by the first shoulder-like absorption structure (Fig. 3(a)), gradually decreases from around 0.4 eV, as clearly illustrated in Fig. 12(a). Right after this field, such structure is absent since the conduction/valence band-edge state near the Fermi level becomes occupied/unoccupied for the **K**/**T** valley. As a result, the threshold frequency, being associated with the excitation of electronic states near the **K** valley, continues to decrease. The further increase of *E*_*z*_ changes the threshold excitation to be near the **T** valley and slightly lowers the optical gap. Regarding the magneto-optical threshold peak in the absence of an electric field, its frequency is characterized by the type-II absorption peak of ${n}_{1\downarrow }^{v}\mathrm{=0}$ → ${n}_{1\downarrow }^{c}\mathrm{=12}$ (red arrow in Fig. 9(a)). The optical gap monotonically grows with the increment of magnetic field, as clearly shown in Fig. 12(b). This is consistent with the *B*_*z*_ evolution of LL energies, in which the conduction/valence LL energies gradually rise/fall with increasing *B*_*z*_ (Fig. 8(a)). Under the composite fields, the threshold frequency has an inverse relation with the electric field for a fixed magnetic field, as demonstrated in Fig. 12(c) for *B*_*z*_ = 40 T. With increasing *E*_*z*_ from zero up to 153 meV/Å, the optical gap in general decreases. For *E*_*z*_ < 106 meV/Å where the band gap is nonzero, the threshold peak is determined by the type-II ${n}_{1\downarrow }^{v}\mathrm{=0}$ → ${n}_{1\downarrow }^{c}\mathrm{=12}$ excitation channel; the optical gap monotonically decreases with *E*_*z*_. After that, the threshold peak relates to the type-I excitation channel of ${n}_{\downarrow 2}^{c}=0$ → ${n}_{\downarrow 2}^{c}=1$ at the **K** valley (red arrow in Fig. 11(a)); the optical gap decreases more quickly (from the green arrow to the purple one). As for even greater *E*_*z*_, the threshold peak corresponds to the type-I excitation channels at the **T** point and its frequency decreases slowly.

There are certain important differences between bilayer AB-bt silicene and AB-stacked graphene in magneto-electronic and optical properties^33^. For the latter, all the LLs possess eight-fold degeneracy without sublattice non-equivalence and spin splitting, mainly owing to the absence of the buckled structure, very significant interlayer hopping integrals, and important SOCs. Their localization centers are only present at 1/6 (4/6) and 2/6 (5/6), but absent at 1/4 (3/4). They are not affected by an electric field. However, the state degeneracy is reduced to half in the presence of *E*_*z*_, since the inversion symmetry/bi-sublattice equivalence is destroyed by the Coulomb potential site energies. The sublattice-dependent LL energy spectra exhibit diverse behaviors,i.e., anti-crossing, crossing & non-crossing behaviors with varied *B*_*z*_/*E*_*z*_. However, there are no low-lying anti-crossing spectra in silicene systems. Considering the first group of valence and conduction LLs, the available magneto-excitation category/categories is/are one/two in the absence/presence of *E*_*z*_. Obviously, the magneto-absorption peaks are well characterized by the specific selection rule of Δ*n* = ±1 except that the extra Δ*n* = 0 & 2 rules might appear under a perpendicular electric field. Their quantum numbers are much smaller than those in bilayer silicene. The threshold channel is only associated with the small quantum-number LLs; that is, it is determined by *n*^*v*^ = 0/1/2 and *n*^*c*^ = 1/0/1.

Absorption^41,42^, transmission^42–45^, and reflection spectroscopies^42,46^ are the most efficient techniques in exploring the essential optical excitations of condensed-matter systems. They are employed as analytical tools for the characterization of optical properties, when the experimental measurements are taken on the fraction related to the adsorbed, transmitted, or reflected light by a sample within a desired frequency range. A broadband light source is utilized and done through a tungsten halogen lamp with the broad range for modulation intensity and frequency^43,46^. The transmission experiments have confirmed that the absorption intensity of monolayer graphene is proportional to the frequency because of the linear dispersions in the isotropic Dirac cone of massless fermions^43^. Besides, massive Dirac fermions are identified in AB-stacked bilayer graphene^42,45^. Moreover, infrared reflection and absorption spectroscopies are also utilized to verify the partially flat and sombrero-shaped energy bands of ABC-stacked few-layer graphene^41^. Three kinds of optical spectroscopies are very useful to examine the stacking- and *E*_*z*_-enriched vertical excitation spectra of AB-bt bilayer silicene, e.g., form, intensity, number and frequency of special absorption structures.

Magnetic quantization phenomena of low-dimensional systems could be investigated using magneto-optical spectroscopies^43,44,47–49^. The magnetic field is presented by a superconducting magnet^43,44,49^ and semi-destructive single-turn coil^47,48^ with the desired field strength below 80 T. The examined/verified phenomena are exclusive in graphene-related systems, such as dispersionless LLs in layered graphene^43,44,49^ and quasi-one-dimensional Landau subbands in bulk graphite^43,44,49^. A lot of pronounced delta-function-like absorption peaks are clearly revealed by the inter-LL excitations arising from massless and massive Dirac fermions in monolayer^43^ and AB-stacked bilayer graphene^49^, respectively. For absorption peak frequencies, the former and the latter obviously exhibit the square-root and linear *B*_*z*_-dependencies. Concerning inter-Landau-subband excitations in Bernal graphite, one could observe a strong dependence on the wave vector *k*_*z*_, which characterizes both kinds of Dirac quasi-particles^47,48^. The rich and unique magneto-optical spectra in bilayer AB-bt silicene are worthy of further experimental examinations, covering diverse absorption structures, *B*_*z*_- and *E*_*z*_-created excitation channels/threshold frequency, many absorption peaks within a very narrow frequency range, and the absence/presence of specific selection rules. They could provide the rather useful information about the buckled structure, stacking configuration/interlayer hopping integrals, and significant layer-dependent SOCs.

## Conclusion

In summary, we have investigated the electric field enriched optical properties of AB-bt bilayer silicene in the absence and presence of a perpendicular magnetic field. An applied electric field creates spin-split states, produces a semiconductor-metal phase transition and forms Dirac cones in different valleys. As a result, some unexpected optical features are detected in the absorption spectra. The external field also leads to drastic changes in the LLs, and thus more complex magneto-absorption spectra. Since the oscillating band structure becomes pronounced, both well-behaved conduction and valence LLs come to exist at each valley. The splitting of sublattice- and localization-dominated LLs become more distinctive. Accordingly, the inter-LL optical transitions at lower frequency with specific selection rules are revealed in the absorption spectra. The threshold frequency gradually declines with increasing *E*_*z*_. The electric field-controlled optical properties open a new opportunity in the application of novel designs of *Si*-based nano-electronics and optical devices with enhanced mobilities.

We combined the generalized tight-binding model with Kubo formula for an efficient scheme to do numerical calculations. This theoretical framework is suitable for full exploration of essential properties of many other emergent 2D materials. The rich and unique critical properties are identified from the special buckled structure, symmetric stacking configuration, complicated intralayer and interlayer atomic interactions, and significant layer-dependent SOCs. They are in sharp contrast with those in layered graphene. Our theoretical predictions could be verified by various optical spectroscopies.
